# Bioglasses as Local Drug Delivery System of Ketoprofen for Regenerative Medicine

**DOI:** 10.3390/ma19071407

**Published:** 2026-04-01

**Authors:** Ruxandra-Elena Geanaliu-Nicolae, Roxana-Cristina Popescu, Paul Emil Mereuță, Voicu Georgeta, Ramona Elena Meja, Ștefan Claudiu Turculeț

**Affiliations:** 1 Department of Biomaterials and Medical Devices, Faculty of Medical Engineering, National University of Science and Technology Politehnica Bucharest, 011061 Bucharest, Romania; 2 Department of Life and Environmental Physics, National Institute for R&D in Physics and Nuclear Engineering-Horia Hulubei, 077125 Măgurele, Romania; 3 Department of Science and Engineering of Oxide Materials and Nanomaterials, Faculty of Chemical Engineering and Biotechnologies, National University of Science and Technology Politehnica Bucharest, 011061 Bucharest, Romania; georgeta.voicu@upb.ro; 4 Department of Surgery, “Carol Davila” University of Medicine and Pharmacy, 8 Eroii Sanitari, Sector 5, 050474 Bucharest, Romania

**Keywords:** bioglass, ketoprofen, tissue ingrowth, local drug delivery

## Abstract

This study explores the potential utilization of bioactive glasses using different dopant ions and ketoprofen for both tissue ingrowth and local drug delivery. Four different compositions of vitreous powders were synthesized by the sol–gel combined with the emulsion method, in the presence of the ionic surfactant cetyltrimethylammonium bromide (CTAB), differing by dopant ions: SiO_2_- P_2_O_5_-CaO-(ZnO-MgO). This study investigates the chemical–mineralogical, morphological, and structural characteristics, as well as the biological properties of vitreous materials obtained. X-ray diffraction (XRD) and Fourier transform infrared spectroscopy (FT-IR) data analysis confirmed the vitreous nature; scanning electron microscopy (SEM) micrographs correlate with the results of physical absorption with N_2_, and the compositions used for the synthesis of the powders all showed for the samples with MgO lower porosity. Biological testing demonstrated biocompatible behavior towards osteoblast cells, (MG-63 type), inducing a slight acceleration of the mineralization phenomenon in the osteoid of the cells compared to the negative control, with cell viability for all the samples higher than 50%. Preliminary release analyses performed by UV–Visible spectroscopy showed a characteristic controlled release profile with prospects for a potential drug delivery system. The zinc–magnesium co-doped sample exhibits optimal performance in both osteogenic promotion and drug delivery, presenting potential for integrated bone repair and local drug administration. This study concludes that the synthesized bioglass exhibits promising characteristics for potential applications in tissue engineering with local drug delivery.

## 1. Introduction

Bioactive glasses are widely investigated for tissue engineering, where high specific surface area and specific porosity enable both tissue ingrowth and local drug delivery.

Bioglass biomaterials are a family of bioactive glasses composed of silicon dioxide, sodium oxide, calcium oxide, and phosphorus pentoxide. These materials exhibit a high specific surface area, a surface pore structure of approximately 5–20 nm, a high pore volume, and good cytocompatibility. Due to these unique properties, clinical applications of bioglass have focused on osteogenesis, as in the case of other biomaterials such as hydroxyapatite or calcium phosphate [1,2]. Due to its ability to form chemical bonds with the surrounding native tissue, this biomaterial not only promotes excellent repair of bone defects but also prevents the stimulation of the formation of an immune fibrous capsule around the implant. Despite these beneficial properties, histological evaluation of long-term implants made of 45S5 Bioglass revealed that it exhibits behaviors of partial degradation, fragmentation, and invasion of the connective tissue of the implant and the implantation area [1].

One of the most studied bioactive glasses for biomedical applications is the commercial 45S5 bioglass. Although it was invented more than half a century ago, it remains a common topic in research aimed at improving the bonding properties of implants to bone tissue. Bioactive glasses are a class of inorganic bioactive ceramics that react with physiological fluids to form strong bonds with bone tissue by forming hydroxyapatite-like layers in bone, as well as through biological interactions between collagen and the material’s surface [3,4,5]. The literature describes how reactions on the surface of bioglass lead to the release of critical concentrations of soluble ions (such as Si, P, Ca, and Na) and thus induce intracellular and extracellular responses favorable to the rapid formation of new bone tissue [3]. Bioglass has emerged as an up-and-coming field of research. An important direction in the study of this biomaterial has been its loading with therapeutic agents to improve tissue regeneration around the damaged area. The loading of bioglass with a drug depends on its crystallinity, as well as the implant’s porosity and morphology, and the release of the active substance is dictated by the degradation rate, which can vary from a few hours to a few weeks. By manipulating this variability, control over the release rate of agents loaded into the material can be achieved. It is known that bioglass degrades through hydrolysis of the silica matrix, leading to the formation of orthosilicic acid and silanols. These processes contribute crucially to the development of new bones by initially increasing the pH at the surface, thereby promoting hydrolytic degradation and nucleation of carbonated hydroxyapatite within the silica gel layer [1].

Bioglass is an amorphous solid material without a precise long-range order, being characterized by the same building blocks (cationic polyhedra) of other similar materials. However, bioglasses present a random arrangement of these elements and wider distributions of bond angles. From a compositional point of view, three main classes of bioglasses can be identified, based on SiO_2_, P_2_O_5_, and P_2_O_3_, with the first being the most studied and most frequently used for biomedical applications [3,4,5,6].

By adding modifying oxides such as alkali or alkaline earth metals to the continuous three-dimensional network interconnected by SiO_2_ groups, the discontinuity of the silicate network is obtained by replacing the Si-BO-Si bonds with Si-NBO bonds, where BO and NBO represent the oxygen atom that forms bonds and the oxygen atom that does not form any bonds. Among the most commonly used modifying oxides are CaO, Na_2_O, and K_2_O. Obtaining this discontinuity allows stabilization of “inverted” glasses containing small amounts of silica, which are characterized by network fragments, possibly interconnected, as in the case of bioactive glasses [7,8].

Dopant ions introduced into the bioactive glass structure enhance the material’s bioactivity or stimulate the desired response in bone tissue. Compared to other types of glass, those with biological activity must have a maximum content of 60% SiO_2_ if obtained by the melting process or a maximum of 85% SiO_2_ in the case of synthesis by the sol–gel method. Otherwise, the bioglasses obtained lose their bioactivity. Similarly, the bioglass must have a high CaO/P_2_O_5_ ratio [8,9,10,11,12,13,14].

The specific properties of bioactive glasses can be improved and controlled when synthesized at the nanoscale. In recent years, there has been a growing interest in nanoscopic silicate bioglasses in the specialized literature, this being due to their superior bioactivity, improved osteoconductivity, and antibacterial properties compared to conventional bioactive bioglasses (of micron size) [1].

Since the original formulation by Hench in the late 1960s, many bioactive glass systems have been established for use in several biomedical applications, including the treatment of osteomyelitis, repair of orthopedic bone defects, bioactive coatings, periodontal reconstruction, or small bone implants [7,15,16,17]. Bioactive glass can be used as a bone graft because it can bond to specific connective tissues by forming collagen on the surface. Bioglass, due to its interconnected porosity, offers advantages for hard tissue implants, as its porous structure supports tissue growth and improves implant stability through biological fixation. The fact that bioglass is a highly effective material for this type of application is confirmed by numerous clinical studies. Among these is the use of bioactive glass particles in the treatment of human periodontal bone defects. In this study, significantly less gingival recession was observed in the bioactive glass-treated areas than in the control areas. Also, greater filling of the defect was observed in regions with bioactive glass, which showed a significant improvement in clinical parameters [1]. Other studies that involved filling periodontal bone defects with bioglass particles demonstrated a substantial increase in the density and radiographic volume of the treated defects, thus demonstrating the potential of this material for treating bone defects.

It is known that bioglass is a material commonly used in studies in bone tissue engineering. One of the main obstacles in the use of bioglass in tissue engineering is the need to imitate the extracellular matrix. Scaffolds made of biocomposite nanofibers and nano-hydroxyapatite were naturally very porous, facilitating cell proliferation, vascularization, nutrient transport, and the removal of metabolic waste. Studies comparing bioinert glass-ceramic scaffolds with highly bioactive glass scaffolds have demonstrated that the latter promote increased proliferation and differentiation of osteoblast cells. Furthermore, some studies indicate that bioglasses induce human fetal osteoblasts to attach, migrate, proliferate, and mineralize into bone, representing a significant step towards filling bone defects [18,19,20,21,22,23].

Also, over the last 20 years, bioactive glass-based systems have offered an alternative for loading and releasing multifunctional therapeutics, with optimized kinetics that can improve drug availability at the target site. Although drug carriers are generally inert, bioglasses are unique among biomaterials in having the potential for pharmacological activity through ionic dissolution products released into the physiological environment, thereby promoting the regeneration of hard and soft tissues [1].

The advantages of using bioactive glass for targeted drug delivery include eliminating many of the drawbacks of systemic treatment. These include frequent administration, peak–plateau effect, interaction with non-target sites, or high drug concentration required to reach the desired site [23].

The antibacterial activity of bioactive glass is due to its properties, such as the ability to increase ambient pH, high osmotic pressure, and mechanical damage to cell walls caused by sharp debris. Through these mechanisms, the use of bioglass can lead to the death of microorganisms. Numerous studies in the specialized literature highlight that the antibacterial effect of bioglass is attributed to its alkaline nature. Most tests demonstrate a considerable reduction in bacterial viability used species such as *Streptococcus sanguis, Streptococcus mutans, or Actinomyces viscous* [7,8,9,10,11].

Since the discovery of bioactive glass more than 50 years ago, many variations in the material composition have been studied and developed to improve the properties of this biomaterial. So far, ions have been studied for doping bioglass to enhance bioactivity, angiogenesis, osteogenic stimulation, and antibacterial effects [8,20,21,22,23].

It is self-evident that understanding and deepening the mechanisms by which dopant ions influence the physical and biological properties of bioactive glasses has become crucial for future research directions and for developing more promising, better-performing biomaterials. Zinc is an essential microelement found in all biological tissues and serves as a cofactor for many enzymes, and also has a necessary role in the growth, development, and differentiation of bone cells. Although the information on the influence of zinc ions on the ability to induce the deposition of a calcium phosphate layer on the surface of bioglass remains contradictory, it is recognized that at concentrations below 10 mol%, Zn exerts a beneficial effect on bioactivity. Also, the larger surface area of the Zn-substituted glass may provide better nucleation sites when immersed in SBF solution, making the calcium phosphate phase more crystalline. Moreover, various studies have shown the osteogenic potential of sol–gel bioglass doped with 5 mol% ZnO, which exhibits enhanced alkaline phosphatase (ALP) activity and osteoblast cell proliferation [7,14,15,16,17,18,19].

Zinc shows strong antibacterial activity against *B. subtilis* and *P. aeruginosa* strains. Zinc ions can be readily incorporated into the bioglass structure and released progressively during dissolution, ensuring controlled, sustained delivery of the antibacterial agent. Zinc also shows promising activity in the treatment of chronic inflammation and processes characterized by an inflammatory component, such as wound healing [24].

Magnesium is the fourth most abundant cation in the human body, being present at a content of 0.44, 1.23 and 0.73% by weight in natural enamel, dentin and bone. Approximately 65% of the total magnesium in the body is found in bones and teeth and plays a role in bone metabolism, promoting new bone formation through direct interaction with integrins on osteoblast cells, which are responsible for cell adhesion and stability [25,26,27,28]. Once introduced into the bioglass network, magnesium ions act as network formers or modifiers. By doping bioactive glass with magnesium, its bioactivity is remarkably improved within just a few days of immersion in simulated body fluid (SBF), due to the increased dissolution rate of the bioactive glass resulting from the disruption of its silica network [7].

Ketoprofen is part of the category of nonsteroidal anti-inflammatory drugs (NSAIDs), representing one of the most used NSAIDs due to the rapidity and effectiveness of its activity. Nonsteroidal anti-inflammatory drugs (NSAIDs) have analgesic, anti-inflammatory, and antipyretic properties, thus reducing fever and pain and preventing inflammation. These types of drugs the advantages of being simple, inexpensive to administer. For example, studies have shown that in the case of surgical intervention such as total hip arthroplasty, treatment with a nonsteroidal anti-inflammatory drug for 14 days postoperatively reduces the occurrence of chronic pain and disability, with a very low risk of adverse effects. NSAIDs also prevent the formation of new bone with ectopic tissue in the soft tissues around the hip joint.

Ketoprofen is a drug indicated for the symptomatic treatment of inflammatory and degenerative rheumatic diseases, as well as for the relief of pain in certain painful syndromes. Some of the main conditions for which ketoprofen is recommended are rheumatoid arthritis, osteoarthritis, seronegative spondyloarthropathies, acute joint or peri-articular diseases, cervical spondylosis, low back pain, post-traumatic joint, muscle or connective tissue pain, and inflammation after orthopedic surgical procedures [29,30,31].

Bioglasses are materials with superior biocompatible properties and can be used in applications that aim to regenerate bone by repairing damaged tissues and stimulating the growth of new bone tissue [24]. These biomaterials have made it possible to move from an intensively studied material in the research field to a significant candidate considered for most clinical applications aimed at the skeletal system, such as the treatment of fractures and defects or bone reconstruction surgery, due to their ability to support bone regeneration and improve its healing process. Also, mesoporous bioactive glasses possess a higher specific surface area and pore volume than conventional bioactive glass. Also, the loading efficiency of drugs and growth factors in MBG is significantly higher, and the drug release kinetics from this material is lower than that of dense bioglass [32,33,34,35,36]. The aim of this project is due to the importance of bioglasses as biomaterials in the field of research and the significant potential for clinical applications. Bioglasses can bring important benefits in the treatment of bone defects, especially in pathological cases such as bone defects or fractures.

The novelty of this study is the combination of sol–gel and emulsion synthesis methods for Zn^2+^/Mg^2+^ doped bioglass as suitable support material for ketoprofen loading with potential use in tissue engineering applications, integrating structural and biological functions and analysis but also preliminary release drug aspects.

## 2. Materials and Methods

### 2.1. Bioglass Synthesis

Based on the literature data [14,15,16,17,18,19], the sol–gel method combined with the emulsion method was used to obtain vitreous masses with potential uses in tissue engineering, including as a drug loading support.

The chosen synthesis method ensured, by using the ionic surfactant CTAB, the obtaining of a vitreous material with a much more advantageous structure for increasing the specific surface area and the capacity to incorporate the active substance ketoprofen.

The choice of oxides used for substitution was made based on their effects in the body [7], namely the improvement of osteogenesis (Mg^2+^) and, respectively, of the anti-inflammatory and antibacterial activity (Zn^2+^). The oxide composition of the 4 samples synthesized in the work is given in Table 1.

For the basic sample, M0, tetraethyl orthosilicate (TEOS, ≥99%, Sigma-Aldrich, Darmstadt, Germany), calcium nitrate tetrahydrate (CN_4_, Ca(NO_3_)_2_·4H_2_O, ≥99%, Sigma-Aldrich, Darmstadt, Germany), and dibasic ammonium, ((NH_4_)_2_HPO_4_, Sigma-Aldrich, Darmstadt, Germany) were used as precursors. In the case of the composition in which the mesoporous bioglass was doped with magnesium, M2, magnesium hexahydrate nitrate (MN_6_, Mg(NO_3_)_2_·6H_2_O, 99%, Sigma-Aldrich, Darmstadt, Germany) was used as a precursor, while for the composition doped with zinc, M1, zinc acetate dihydrate (ZnAc_2_ Zn(CH_3_COO)_2_·2H_2_O, ≥98%, Sigma-Aldrich, Darmstadt, Germany) was used. In the case of the last composition, M3, mesoporous bioglass with both magnesium and zinc, both previously mentioned precursors were used.

Once the last precursor was added (depending on each composition), the solutions were homogenized for 4 h by magnetic stirring. Following this step, white precipitates were formed. These were centrifuged at 7000 rpm for 5 min, washed repeatedly with distilled water, and then dried at 60 °C. The last step of this process was the calcination of the dried precipitates at 600 °C for 3 h at a rate of 2 °C/min, as determined by differential thermal analysis.

**Figure 1:**
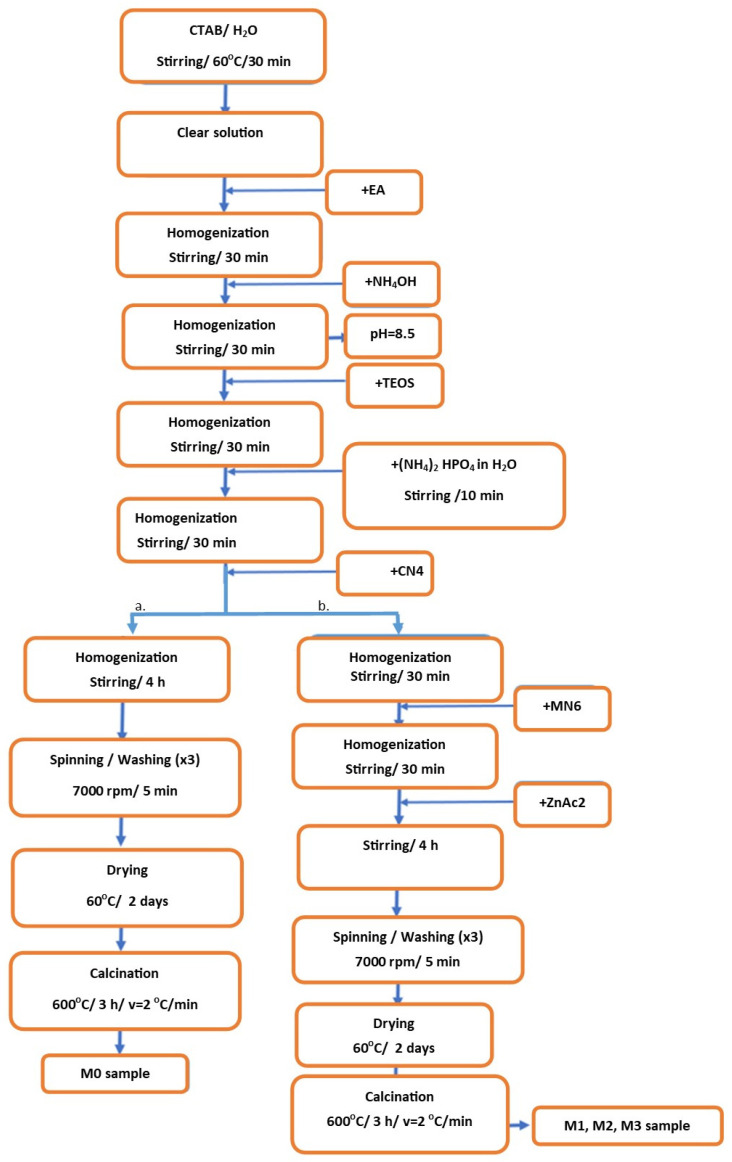
Synthesis process (a). M0 sample, (b). M1, M2, M3 samples.

The steps of the sol–gel method combined with the emulsion method can also be observed in the synthesis schemes presented in Figure 1a, corresponding to the glassy powder without dopant (M0), and Figure 1b, corresponding to the glassy powders with dopants (M1, M2, M3).

### 2.2. Materials Characterization

#### 2.2.1. Thermal Analysis

Thermal analysis was performed in order to determine the calcination temperature of dry precipitates. This analysis was performed using a DTG-TA-60 derivatograph (Shimadzu, Kyoto, Japan) in an oxidizing atmosphere and platinum crucible, falling within the temperature range of 20–1000 °C and having a heating rate of 10 °C/min.

#### 2.2.2. X-Ray Diffraction (XRD)

X-ray diffraction was used to determine the component mineralogical phases, but also to obtain information about the structure of the synthesized material. XRD analysis was performed on dry precipitate samples using a Shimadzu XRD 6000 diffractometer (Shimadzu, Kyoto, Japan) using nickel-filtered copper Kα radiation, a wavelength of λ = 1.5406 Å, a scan speed of 2°/min and a scan step of 0.02°.

#### 2.2.3. Fourier Transform Infrared Spectroscopy (FT-IR)

FT-IR spectroscopy was applied to identify the nature of the bonds that exist in the synthesized glassy powders, and the analysis was performed using a Nicolet iS 50 R spectrophotometer (Thermo Fischer Scientific, Waltham, MA, USA) with attenuation total reflection (ATR) mode, the spectrum being recorded in the range 4000–400 cm^−1^ with a resolution of 2 cm^−1^.

#### 2.2.4. Brunauer–Emmett–Teller (BET) and Barrett-Joyner-Halenda (BJH)

Brunauer–Emmett–Teller analysis involves the precise evaluation of the specific surface area of a sample by measuring the multilayer adsorption of nitrogen as a function of relative pressure. It can also be used to determine the total specific surface area (in m^2^/g) by surface and pore analysis. In addition to the specific surface area, BET can also provide information about the particle size and surface porosity of the analyzed material [38].

Barrett–Joyner–Halenda analysis is a method that uses adsorption and desorption techniques to determine the pore areas and their specific volume. This technique can characterize the pore size distribution of a material independent of the external area due to the particle size of the sample [38].

In this project, Brunauer–Emmett–Teller and Barrett–Joyner–Halenda analyses were used to determine the specific surface area of the synthesized samples, as well as the average size and volume of the pores present in them.

#### 2.2.5. Scanning Electron Microscopy (SEM)

Scanning electron microscopy was used to characterize the microstructure and morphology of glassy powders obtained by the sol–gel method combined with the emulsion method, as well as to determine the size of the particles and pores existing in the material. The analysis was performed using an Inspect F50 microscope (Thermo Fisher-former FEI, Eindhoven, The Netherlands), coupled with an EDX probe.

#### 2.2.6. In Vitro Biological Tests

The tetrazolium salt reduction test (MTT) is a colorimetric method that helps determine cell viability. The principle of this method consists in measuring cellular metabolic activity using cellular oxidoreductase enzymes, cell viability being directly proportional to this metabolic activity. They can reduce the yellow tetrazolium salt, which is soluble, to purple and insoluble formazan crystals [39].

The lactate dehydrogenase (LDH) test is a rapid technique for measuring membrane integrity because lactate dehydrogenase is an enzyme used as an indicator of cell death. This type of test determines the degree of cytotoxicity as a result of the release of the enzyme into the culture medium after damage to the cell membrane [39].

The Alizarin Red cell differentiation assay is a method for detecting and quantifying mineralization, involving the measurement of calcium deposits formed. Alizarin Red is a sodium salt derived from anthraquinone, and the principle of the method is based on its ability to react more specifically with calcium cations through a chelation process [37,39,40].

Osteoblast-like MG-63 cells (CLS, Heidelberg, Germany) were cultured in Dulbecco’s Modified Eagle Medium (DMEM) supplemented with 10% fetal bovine serum and 1% Penicillin-Streptomycin under standard temperature and humidity conditions (37 °C, 5% CO_2_, 90% humidity).

Samples were sterilized by exposure to UV radiation for 1 h on each side.

Cells were seeded at a concentration of 100,000 cells/100 µL/sample in a 24-well plate and incubated under standard temperature and humidity conditions for 30 min to allow for attachment. After this period, the culture medium was made up to 1 mL, and the cells were incubated under standard conditions for 3 days.

After the incubation period, the culture medium was removed, the samples were washed with PBS and fixed in 2.5% glutaraldehyde for 1 h. Then, the cells were washed with PBS and incubated with increasing concentrations of ethanol solutions (70–100%) for 30 min, followed by incubation with HMDS: ethanol solutions, concentrations 50:50, 75:25, and 100% HMDS, respectively.

Microscopy images of the samples used for biological tests were acquired using a Zeiss EVO MA12 microscope (Zeiss, Oberkochen, Germany), with a resolution of 4 nm at 30 kV.

### 2.3. Ketoprofen Bioglasses Synthesis and Release

Starting from the advantages of the synthesis method in inducing open porosities in the vitreous material particle, its capacity to retain and release the drug was studied.

#### 2.3.1. Drug Loading

Injectable ampoules (2 mL ampoule contains 100 mg of active substance), ketoprofen, an anti-inflammatory drug, were used to load M0–M3 samples. The determined amount of synthesized vitreous powder is left under vacuum for 15 min. The powder, together with the drug ketoprofen, is subjected to a new vacuum process lasting 3 h and 30 min. Acetone is added to the obtained samples and left for 1 h and 30 min. Then, the powders were either air-dried for 7 days (RM0, RM1, RM2, RM3) or lyophilized for 24 h (RM0-L, RM1-L, RM2-L, RM3-L).

#### 2.3.2. Preliminary Release Study, UV-Vis Spectrometry

A preliminary in vitro drug release testing of synthesized materials was performed. A total of 0.07 g of non-lyophilized sample and 0.015 g of lyophilized sample were placed separately in 10 mL SBF solution. Aliquots of 1 mL were acquired at different times over 6 days (i.e., 10 min, 30 min, 120 min, 240 min, 1440 min, 2880 min, 4320 min, and 9600 min). The amount of ketoprofen released in the SBF was quantified based on the Beer–Lambert law by a UV–Visible spectrophotometer at absorbance maximum peak for ketoprofen [34,41].

## 3. Results and Discussions

### 3.1. Characterization of the Dried and Thermally Treated Powders

**Figure 2:**
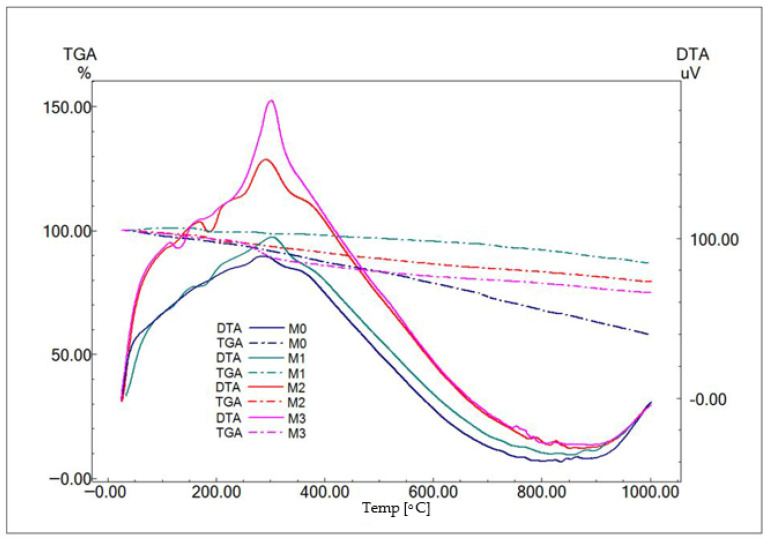
Thermal analysis of dried and thermally treated powders.

**Figure 3:**
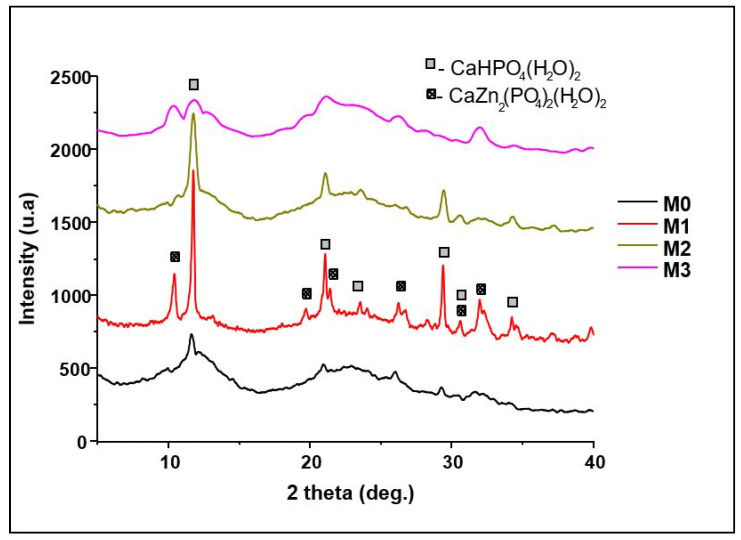
X-ray diffraction pattern of dried and thermally treated powders.

The resulting dried powders were characterized from a compositional point of view by complex thermal analysis—Figure 2, and X-ray diffraction—Figure 3. It can be observed that the thermal analysis differential (ATD) curves highlight an important, exothermic effect at approximately 300 °C accompanied by mass loss that can be associated with the combustion of the residual organic component and endothermic cascade effects, between 120 and 200 °C, of lower intensity, which can be attributed to the decomposition of the two crystal hydrates highlighted by X-ray diffraction–CaHPO_4_(H_2_O)_2_ (PDF 072-0713) and CaZn(PO_4_)(H_2_O)_2_ (PDF 035-0495).

**Figure 4:**
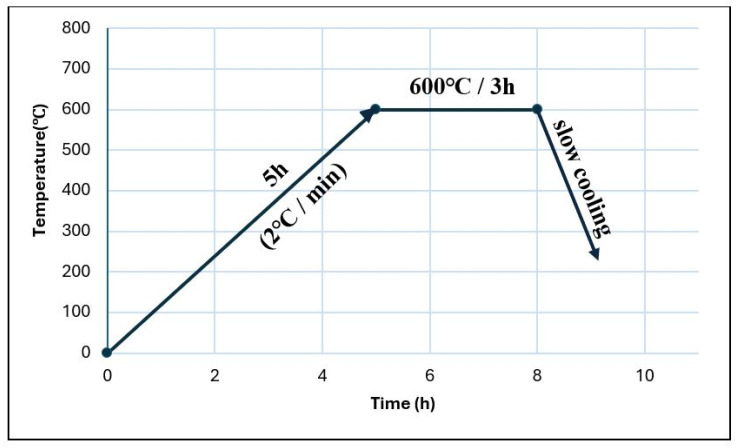
Heat treatment curve applied.

After 600 °C, mass loss is no longer important, which caused the samples to be calcined at 600 °C/3 h, the calcination curve being given in Figure 4, so as to favor the appearance of small open pores.

**Figure 5:**
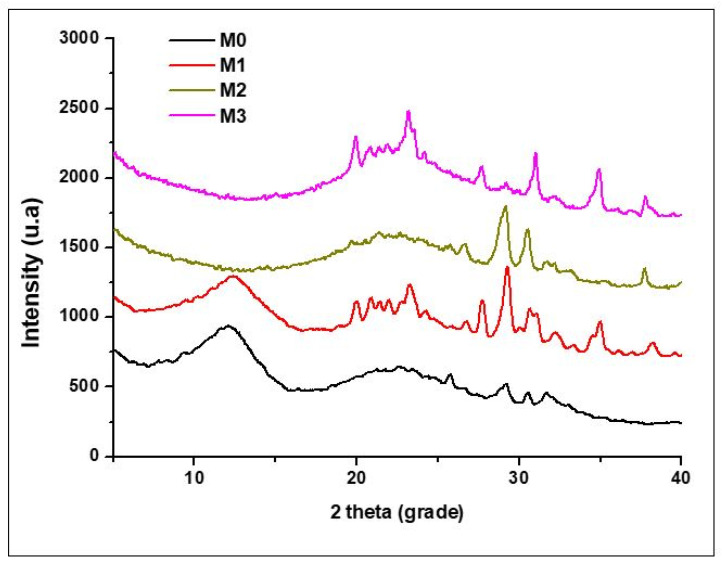
X-ray diffraction pattern for powder calcined at 600 °C/3 h.

**Figure 6:**
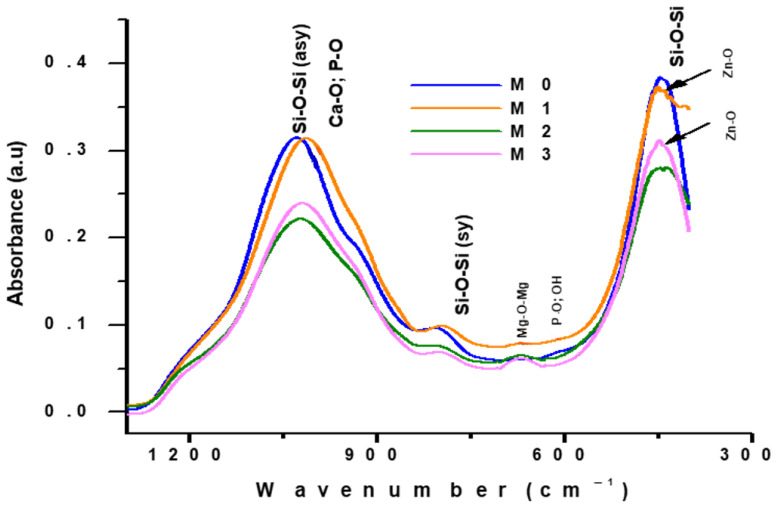
FTIR spectra of calcined powder at 600 °C/3 h.

After calcination heat treatment at 600 °C/3 h, the diffractograms presented in Figure 5 show a low degree of crystallinity, through the presence of diffraction halos in which low intensity characteristic Ca_3_(PO_4_)_2_ (PDF 009-0169) diffraction interferences are found, and the FTIR spectra in Figure 6 show vibration bands characteristic of Si-O-Si at 445 cm^−1^ and 808 cm^−1^ and 1060 cm^−1^ and 602 cm^−1^ for Ca-O and P-O bonds, for all masses, respectively Zn-O- for M1 and M3, and O-Mg-O- for M2 and M3 [42].

**Figure 7:**
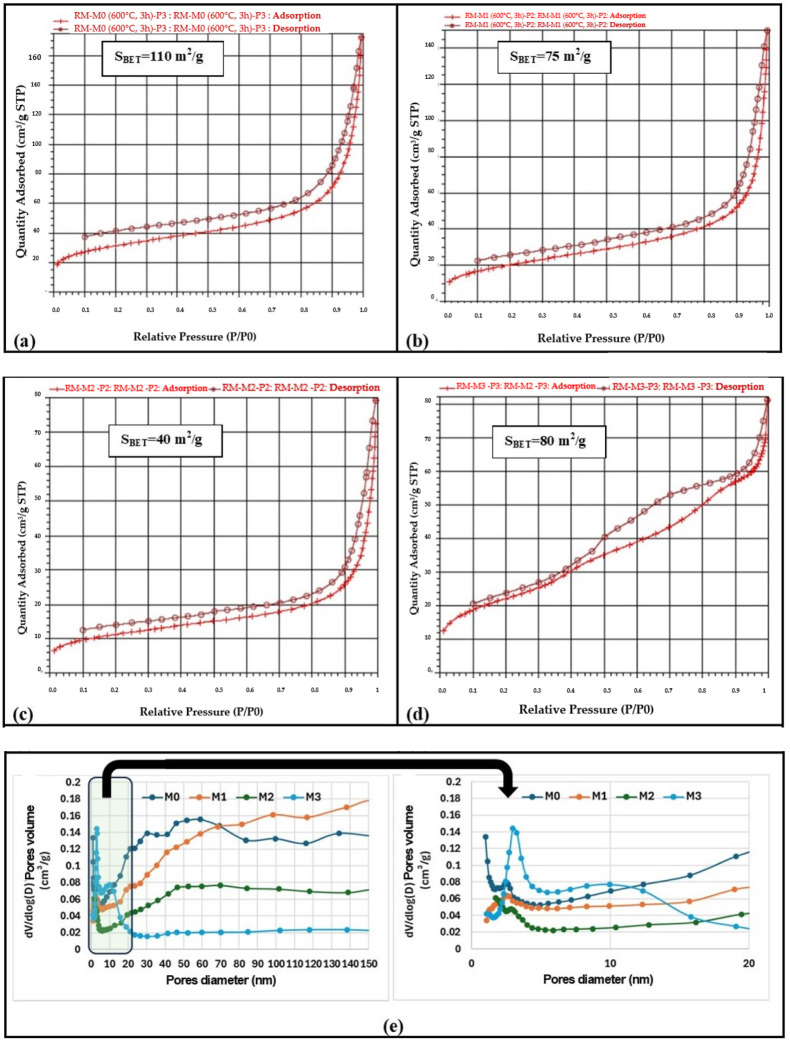
BET analysis performed on powders calcined at 600 °C/3 h-N_2_ adsorption isotherms ((**a**)—M0; (**b**)—M1, (**c**)—M2, (**d**)—M3) and pore distribution (**e**).

In terms of specific surface area and structural characteristics–open porosity, the powders were characterized by BET analysis (N_2_ absorption isotherms), the results being centralized in Figure 7. It can be observed that the isotherms are of type IV, characteristic of porous materials, the specific surface area decreasing in the series M0 > M3 > M1 > M2, directly proportional to the porosity—Figure 7; M0, M1 and M2 powders are characterized mainly by pores with a size above 50 nm (M0 > M1 > M2, which explains the larger volume of adsorbed N_2_). It is also observed that a more important porosity with a size below 20 nm, characteristic of mesopores, is shown by the M3 > M0 > M1 > M2 samples (Figure 7e detail).

**Figure 8:**
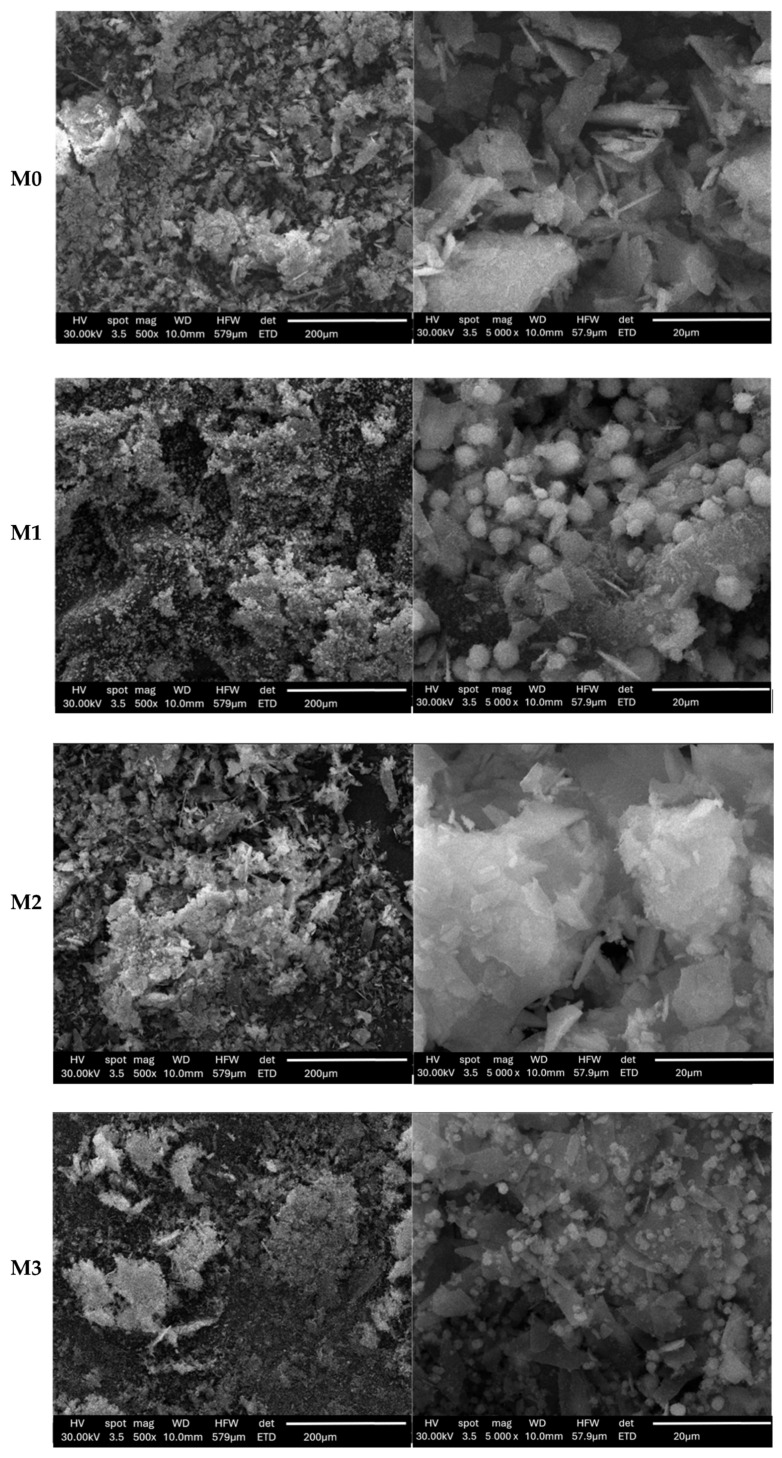
SEM images of powders calcined at 600 °C/3 h.

Morpho-structural characteristics, the calcined powders were analyzed by scanning electron microscopy—Figure 8, noting that the obtained vitreous materials show dimensional inhomogeneity, having a multimodal distribution. Also, it can be observed that there is a tendency for particles to agglomerate in the case of all four powder compositions. Moreover, it can be noted that there is porosity both at their surface and in the depth of the material, with intergranular spaces present. The SEM micrographs are consistent with the results obtained using the N_2_ physical adsorption/desorption analysis, noting that the M0, M1 and M3 samples show high porosity, while in the case of the M2 powder composition the porosity is lower.

Regarding morphology, it can be noted that the samples show particles with different morphologies depending on the composition used. Thus, in the samples in which the dopant oxide ZnO is present, namely M1 and M3, the presence of quasi-spherical particles is observed, while sample M2, which contained MgO as the dopant oxide, is characterized mainly by polyhedral shaped granules. It can also be noted that in the case of all samples, particles with polyhedral or non-uniform morphology are also distinguished.

### 3.2. Biological Characterization of Synthesized Vitreous Powders

From a biological point of view, the masses were characterized in vitro by incubation with MG-63 osteoblast-type cells to determine their bioactivity and cytotoxicity.

The results obtained for the MTT test after 14 days of incubation were different. Samples M0, M1, and M3 show biocompatibility behavior, with cell viability being around 80% [39], while M2 showed a reduction in cell metabolism up to cell viability values of 50% [39].

**Figure 9:**
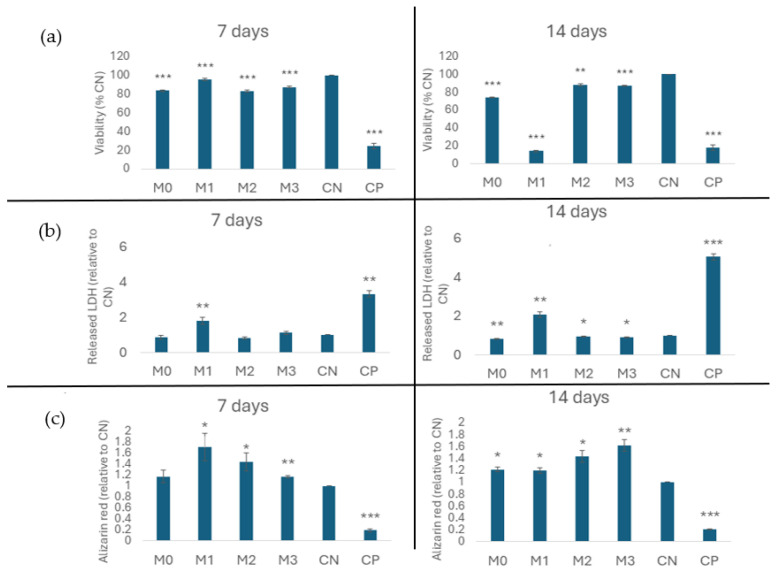
The effect of cell death by necrosis, associated with the destruction of the cell membrane in vitro tests by incubation with osteoblast-type MG-63 cells, at 7 and 14 days respectively: (**a**)—MTT, (**b**)—LDH, (**c**)—cell differentiation with Alizarin Red (* *p* < 0.05, ** *p* ≤ 0.01 and *** *p* ≤ 0.001).

Results of the LDH test (Figure 9), which characterizes the effect of cell death by necrosis, associated with the destruction of the cell membrane, showed that after 7 days of incubation, samples M0 and M2 did not show an increase in the amount of LDH in the extracellular environment following exposure of osteoblast cells to them. Practically, in the case of samples M0 and M2, the calculated relative values were lower compared to the negative control (NC), which can be associated with a reduction in the number of cells in the respective sample, compared to the negative control. It is also observed that the cells exposed to samples M1 and M3 suffered cell membrane damage, measured by the release of LDH into the extracellular medium, to a small extent (compared to the negative control). The results obtained after 14 days of incubation are like those obtained after 7 days.

The results of the cytotoxicity tests suggest the following conclusions. The biocompatibility of samples M0 and M2 is observed. Sample M2 shows biocompatible behavior at 7 and 14 days, manifested by the fact that LDH was not released into the extracellular environment (relative to the negative control); cellular metabolism is maintained within the limits of biocompatibility at 7 days of cultivation, but after 14 days a slight decrease is recorded up to 50%.

Sample M1 induces a cytotoxic effect on osteoblast-type cells, manifested by the release of LDH, but the active maintenance of metabolism at 7 days of cultivation, respectively by the significant reduction of cellular metabolism associated with the release of LDH into the extracellular environment after 14 days of cultivation. The mechanism of cell death is associated with a necrotic phenomenon. Samples M1 and M3 show a slight cytotoxic effect associated with the release of LDH enzyme from osteoblast cells into the extracellular environment after 7 and 14 days of cultivation, a phenomenon associated with maintaining cellular metabolism within biocompatibility limits [39]. The cell differentiation test with Alizarin Red (Figure 9c), which involves measuring Ca deposits formed following osteoid mineralization, showed that after 7 days of incubation, a significant increase in mineralization is revealed in osteoblast cells exposed to samples M0 and M3, compared to the negative control. The results after 14 days of incubation reveal a significant increase in mineralization in osteoblast cells exposed to samples M1, M2, M3.

In conclusion, samples M0 and M1 demonstrate biocompatibility towards osteoblast cells, inducing a slight acceleration of the mineralization phenomenon in the osteoid of the cells compared to the negative control after 7 days of incubation, an effect that is, however, lost after 14 days. Cell morphology investigations support these statements. Sample M2 demonstrates biocompatibility towards osteoblast cells, inducing a slight acceleration of the mineralization phenomenon in the osteoid of the cells after 14 days, compared to the negative control. Cell morphology investigations support these statements. Sample M3 demonstrates a slight cytotoxic phenomenon manifested by the release of LDH into the culture medium. The sample induces a slight acceleration of the mineralization phenomenon in the osteoid of the cells after 14 days, compared to the negative control. The reduction in cellular metabolism is associated with the phenomenon of cellular differentiation. Cell morphology investigations support these statements.

**Figure 10:**
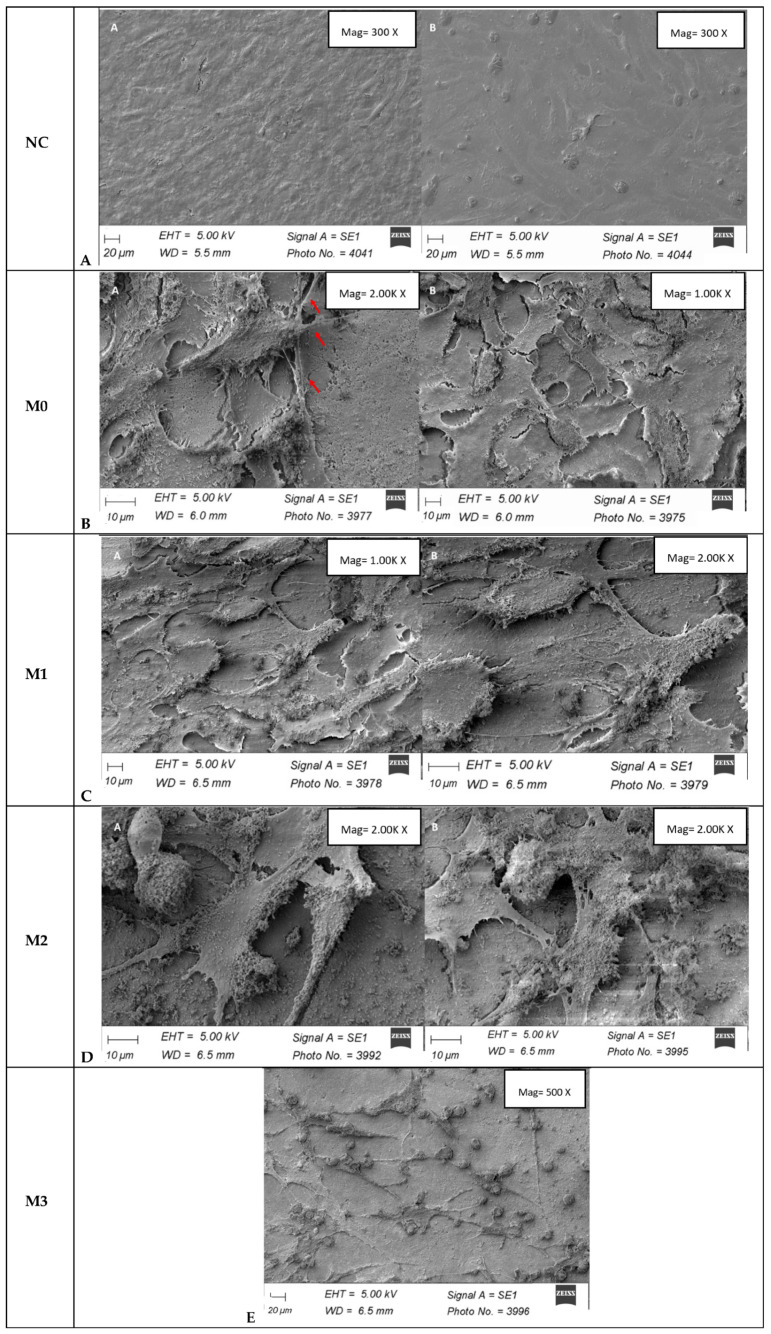
SEM image of MG63 osteoblast cells cultured for 3 days under standard conditions on synthesized vitreous masses. Red arrows show adherence points of the osteoblast cells. ((**A**)—NC, (**B**)—M0, (**C**)—M1, (**D**)—M2, (**E**)—M3).

SEM images for MG63 osteoblast cells cultured for 3 days under standard conditions on the negative control and M0–M3 sample are given in Figure 10.

The negative control is represented by osteoblast-type cells adhered to the glass. Their elongated and flat morphology is observed under super-confluent conditions.

The SEM image of MG63 osteoblast cells cultured for 3 days under standard conditions on the M0 sample showed good adhesion of the osteoblast cells at confluence. They have polygonal morphology but are not completely spread out to the substrate, probably due to the rough appearance of the M0 ceramic surface. The cells show adhesion points in the form of dendritic-like extensions, suggesting that is initiating a differentiation process.

For sample M1, good adhesion of osteoblast-type cells, at confluence, is observed. Although the cells have a polygonal morphology, their tendency to elongate and orient in a certain direction can be observed. Multiple cytoplasmic extensions (filopodia) with an adhesion role are observed.

For the M2 sample, good adhesion of osteoblast-type cells is observed. The cell density is lower in this sample compared to CN, M0 or M1. The cells have a stellate morphology, with multiple cytoplasmic extensions that play a role in adhesion. The cells follow the irregular surface of the material, so they do not present a flat, spread-out appearance.

For sample M3, a reduced density of osteoblast-type cells is observed, compared to the rest of the samples. Some of the cells have an elongated morphology, are exposed to the substrate and present multiple cytoplasmic extensions, but there are multiple rounded cells.

### 3.3. Characterization of Synthesized Vitreous Powders for a Potential Drug Release Support

**Figure 11:**
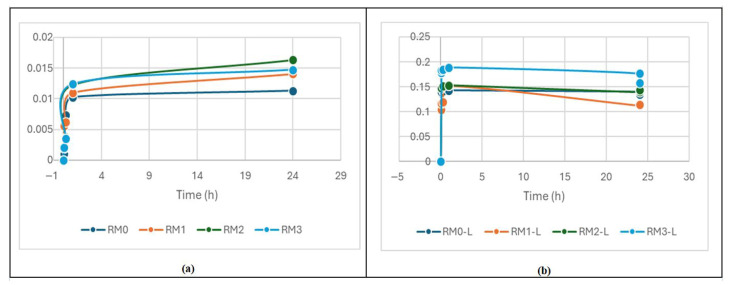
Controlled release results for air-dried (**a**) and lyophilized (**b**) samples.

A specific graphical pattern represents the incipient kinetics study of drug release in a controlled-release system. The releases were determined at 233 nm (Ketoprofen British Pharmacopoeia Monograph). Primary graphs were obtained by UV spectrometry to follow the kinetics of ketoprofen release from experimentally prepared vitreous materials and, ultimately, to establish, in perspective, a possible mechanism of its release. After processing the experimental data (Figure 11) and the quantitative evaluation of ketoprofen released from the samples, it can be observed that in the first 4 h, there is a rapid increase in the amount of drug released from all materials, followed by a slow release over the next 160 h. Compared to the non-lyophilized samples, the amount of drug released from the lyophilized samples is higher, likely due to the presence of propylene glycol in the initial, non-lyophilized samples. For lyophilized samples, although drug diffusion is observed, erosion-controlled is also present. For non-lyophilized samples the release is more gradual and does not present that instantaneous burst in the first 5 min as for lyophilized samples. Also, for non-lyophilized samples the release is dominated by simple diffusion, following the Higuchi model that can be further analyzed. The actual data shows some variability, and RM1 is the sample that most closely follows a standard mathematical model of release, presenting the more stable release.

However, the release graph is specific to controlled release for both air-dried and lyophilized samples, demonstrating the achievement of the proposed release system, with promising data for mathematical models of release.

## 4. Conclusions

In this study, four different compositions of vitreous powders were synthesized by the sol–gel method combined with the emulsion method and in the presence of the ionic surfactant CTAB, which varied by the presence of specific dopant ions, SiO_2_- P_2_O_5_-CaO-(ZnO-MgO). The chosen synthesis method, using the ionic surfactant CTAB, ensured the production of bioglass material with an increased specific surface area and the possibility of incorporating drugs such as ketoprofen [1]. The choice of oxides used for substitution was based on their effects in the body [2], with Mg^2+^ for the improvement of osteogenesis and for anti-inflammatory activity and Zn^2+^ for antibacterial activity. To achieve this objective, this study investigates the chemical–mineralogical, morphological, and structural characteristics, as well as the biological properties, of vitreous materials obtained by the sol–gel method combined with the emulsion method. XRD and FT-IR data analysis confirmed the vitreous nature of the samples, showing halos and a low degree of crystallinity, as well as the presence of bonds specific to silicate glasses. The SEM micrographs are in correlation with the results of physical absorption with N_2_ and the compositions used for the synthesis of the powders, observing that sample with MgO presents a lower porosity than the others, but also different morphology of the grains existing in the material depending by the presence of dopant oxides—quasi-spherical particles in samples with ZnO, polyhedral particles in samples with MgO content. Biological properties of the synthesized vitreous powders showed biocompatible behaviors. The MTT test shows that after 7 days of incubation, cell viability exceeds 80%. LDH and Alizarin Red tests were performed, and as a result, a slight acceleration of the mineralization phenomenon in cellular osteoid for all compositions and a reduction in cellular metabolism, associated with the phenomenon of cellular differentiation, could be found in the case of samples containing MgO. These results are in correlation with the SEM micrographs made for the MG63 osteoblast-type cells, for which it was observed that the cells show good adhesion to the substrate, have a polygonal morphology, and reach confluence. The release kinetics of the anti-inflammatory drug, ketoprofen, from the synthesized bioglasses were determined in SBF for 160 h. Each bioglass presents a characteristic graph of controlled releases, both in the case of air-dried and lyophilized samples. This study concludes that the synthesized bioglass exhibits promising characteristics for potential applications in tissue engineering with local drug delivery.

## Figures and Tables

**Table 1:** Bioglass oxide composition (wt%) [37].

Sample	SiO_2_	CaO	P_2_O_5_	ZnO	MgO
M0	68	28	4	-	-
M1	65	26	4	5	-
M2	65	26	4	-	5
M3	65	26	4	2.5	2.5

## Data Availability

The original contributions presented in this study are included in the article. Further inquiries can be directed to the corresponding author.
